# Supplementary figures and images for: Evaluation potential of PGPR to protect tomato against Fusarium wilt and promote plant growth
Source: PeerJ. 2021 Apr 16;9:e11194. doi: 10.7717/peerj.11194 (PMC8054735; doi:10.7717/peerj.11194)

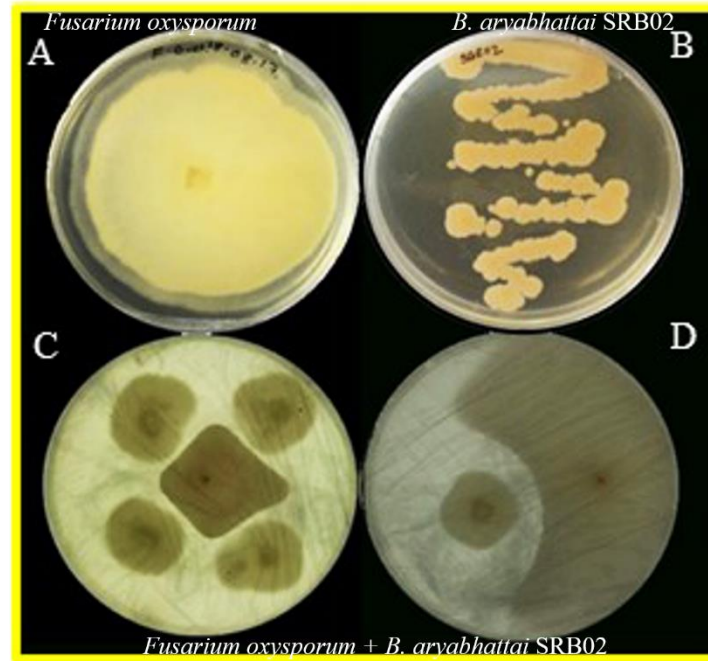

Supplement: Supplemental Information 4 — (A) Fusariumoxysporum f. sp. lycopersici., (B) B. aryabhattai SRB02 (C, D) Growth inhibition of B. aryabhattai SRB02 against Fusarium oxysporum f. sp. lycopersici. [file peerj-09-11194-s004.pdf]
